# How to differentiate behavioral variant frontotemporal dementia from primary psychiatric disorders: practical aspects for the clinician

**DOI:** 10.1590/0004-282X-ANP-2022-S140

**Published:** 2022-08-12

**Authors:** Leandro Boson Gambogi, Leonardo Cruz de Souza, Paulo Caramelli

**Affiliations:** 1 Universidade Federal de Minas Gerais, Faculdade de Medicina, Grupo de Neurologia Cognitiva e Comportamental, Belo Horizonte MG, Brazil.; 2 Universidade Federal de Minas Gerais, Programa de Pós-Graduação em Neurociências, Belo Horizonte MG, Brazil.

**Keywords:** Frontotemporal Dementia, Mental Disorders, Demência Frontotemporal, Transtornos Mentais

## Abstract

**Background:**

Due to the early and prominent behavioral changes which characterize behavioral variant frontotemporal dementia (bvFTD), patients are more likely to seek psychiatric help and are often initially diagnosed with a primary psychiatric disorder (PPD). Differentiating these conditions is critical because of the dramatically different outcomes, differences in patient management, family counseling and caregiver education.

**Objective:**

To propose a practical guide to distinguish between bvFTD and PDD.

**Methods:**

We conducted a non-systematic review of the published manuscripts in the field, including some previous investigations from our own group and work on which we have collaborated, and summarized the main findings and proposals that may be useful for neurological practice.

**Results:**

The reviewed literature suggests that a comprehensive clinical history, brief cognitive and neuropsychological evaluations, detailed neurological examination with special attention to motor alterations related to bvFTD, structural and functional neuroimaging evaluation, genetic investigation in selected cases, and assistance from a multidisciplinary team, including a neurologist and a psychiatrist with expertise in bvFTD, are very helpful in differentiating these conditions.

**Conclusions:**

Although the clinician may commonly face great difficulty in differentiating between bvFTD and PPD, the use of appropriate tools in a systematic way and the availability of a well-trained multidisciplinary group can significantly increase diagnostic accuracy.

## RESUMO

**Antecedentes:**

Devido às alterações comportamentais precoces e proeminentes que caracterizam a variante comportamental da demência frontotemporal (DFTvc), os pacientes são mais propensos a procurar atendimento psiquiátrico e muitas vezes são diagnosticados inicialmente com um transtorno psiquiátrico primário (TPP). Diferenciar essas condições é fundamental, devido aos desfechos significativamente diferentes, diferenças no manejo dos pacientes, no aconselhamento familiar e na educação dos cuidadores.

**Objetivo:**

Propor um guia prático para o diagnóstico diferencial entre DFTvc e TPP.

**Métodos:**

Revisão não sistemática dos manuscritos publicados na área, incluindo algumas investigações anteriores de nosso próprio grupo e trabalhos com os quais colaboramos. Os principais achados e propostas foram sintetizados para auxiliarem a prática neurológica.

**Resultados:**

A literatura revisada sugere que a história clínica abrangente, avaliação cognitiva breve e avaliação neuropsicológica, exame neurológico detalhado com especial atenção às alterações motoras relacionadas à DFTvc, exames de neuroimagem estrutural e funcional, investigação genética em casos selecionados e o atendimento por equipe multidisciplinar, incluindo um neurologista e um psiquiatra com experiência em DFTvc, são bastante úteis na diferenciação dessas condições.

**Conclusões:**

Embora o clínico possa enfrentar grande dificuldade para diferenciar DFTvc e TPP, o uso de ferramentas apropriadas de forma sistemática e a disponibilidade de uma equipe multidisciplinar bem treinada podem aumentar significativamente a acurácia diagnóstica.

## INTRODUCTION

Frontotemporal dementia (FTD) is a clinically, neuroanatomically, and pathologically heterogeneous group of neurodegenerative diseases that share a propensity to target the frontal and temporal lobes of the brain. The FTD spectrum encompasses clinical/anatomical variant syndromes, classified according to their leading features^1^.

The most common FTD phenotype is the behavioral variant frontotemporal dementia (bvFTD), characterized by prominent behavioral changes, with a disruption of multiple domains related to social cognition. The patients manifest neuropsychiatric symptoms that include disinhibition, apathy, hyperphagia or appetite changes, loss of empathy, and impairment in judgment and discernment. In addition, deficits in executive functioning such as perseverative behaviors and difficulties with planning, organizing, and task switching are often seen^2^. Not infrequently, these symptoms are confused with primary psychiatric disorders (PPD)^3^. 

Differentiating bvFTD from primary PPD is critical because of the dramatically different outcomes, differences in patient management, family counseling and caregiver education^3^. There is also a need to accurately identify patients with bvFTD in the early stages for future studies with disease-modifying treatments. Relatives of patients with bvFTD usually identify an inaccurate diagnosis as the major issues they experience^4^. 

The purpose of the present work is to guide the clinician to a better assessment of the patient to improve the distinction of bvFTD from primary psychiatric disorders, in particular to pursue an earlier and more accurate diagnosis.

## DIAGNOSTIC CRITERIA AND CLINICAL PICTURE

According to current recommendations, a bvFTD phenotype can be verified if progressive deterioration of behavior and/or cognition is found, in the presence of at least three out of six possible core symptoms, including five behavioral domains: 1) social disinhibition; 2) apathy 3) loss of sympathy or empathy; 4) perseverative, stereotyped, or compulsive behaviors; 5) hyperorality or dietary changes, and a sixth manifestation, represented by a neuropsychological profile characterized by executive dysfunction, with relative preservation of episodic memory and visual/spatial skills. The diagnosis is given as probable when frontal and/or anterior temporal atrophy/hypoperfusion/hypometabolism is evidenced on structural or functional neuroimaging, associated with functional (i.e., daily life activities) impairment^5^. 

Disinhibition includes socially inappropriate behaviors, such as invasion of interpersonal space or excessive familiarity with strangers. There may be impulsive or careless actions, such as gambling addiction, theft and robbery, and inappropriate decision-making without regard to consequences. Loss of social decorum is also common in bvFTD. Patients may tell inappropriate jokes and use foul language without any embarrassment^6^ ^,^ ^7^. 

Lack of empathy or sympathy is frequent. For example, a patient with bvFTD gives an inappropriate response to a family member who has been diagnosed with a serious medical condition. Other manifestations that fit this question include insensitivity and lack of interest for others^6^ ^,^ ^7^.

Apathy manifests as indifference or lack of interest and reduced overall motor and voluntary movement. Patients exhibit a loss of desire to engage in goal-oriented activities, and have social withdrawal in work, family, or hobby activities. Patients often need to be encouraged to stay engaged in conversations, to do household chores, or even to move around. The clinical picture is commonly misinterpreted as depression^6^ ^,^ ^7^.

Perseverative, stereotyped, or compulsive behaviors may also occur in bvFTD. Simple, repetitive motor displays include clapping, rubbing, poking, and lip-snapping. More complex behaviors may be the collection of cigarette butts, counting rituals, or repetitive trips to the bathroom. Speech may also become stereotyped with specific repetitive patterns^6^ ^,^ ^7^. 

Hyperorality and major changes in eating habits can also manifest in bvFTD. Usually, the changes in eating behavior and preferences involve an inclination for sweets or carbohydrates and excessive eating, even when satiated. As patients become more uninhibited, they may take food from other people’s plates. Later, hyperorality may occur, with oral exploration of inedible objects^6^ ^,^ ^7^. 

## EPIDEMIOLOGY

The incidence of FTD is estimated to be 1.61 to 4.1 cases per 100,000 people annually. FTD is the second most common dementia in people under 65 years of age, only after Alzheimer's disease (AD)^8^. There are very few studies on the prevalence of FTD in Latin American countries, but data suggest a prevalence of 1.2 to 1.7 per 1,000^9^.

Men and women are equally affected, and the mean age of onset is between 45 and 65 years. However, there are documented cases under the age of 30, and today it is recognized that up to 30% of patients with FTD have senile onset (i.e., 65 or more years). Among the clinical presentations, the behavioral variant is the most frequent and accounts for about 60% of cases^8^. Another rather common clinical presentation of FTD is characterized by language disturbances (i.e., primary progressive phasias), which are not included in this review.

It is believed that FTD is underdiagnosed among non-specialists, probably due to lack of knowledge or experience, and to the difficulty faced with the overlap of symptoms with several primary psychiatric disorders.

## CLINICAL ASSESSMENT

### Clinical history

The first recommendation is to collect a detailed clinical history, with reserved importance given to establishing a very definite timeline, from the first symptoms to the present day. PPD typically emerges in late adolescence and early adulthood. Severe psychiatric disorders that begin late in life are more atypical and suggest a risk of dementia, warranting more careful investigation^10^ ^,^ ^11^. 

The review of the previous medical history should include a thorough investigation of psychiatric presentations such as depressive, anxious, psychotic, hypomanic, and manic symptoms. The clinician must also investigate substance abuse, neurodevelopmental disorders, suicide attempts, attention deficit hyperactivity disorder (ADHD), and personality traits. Also, well-known risk factors for dementia need to be investigated, such as cerebrovascular disease and traumatic brain injuries^3^. Medications in use and any current substance use must be described. 

Information regarding family history of PPD, dementia and motor neuron disease is critical, but there are caveats, as the presence of a positive family history for psychiatric disorders tends to miss a diagnosis of bvFTD. In some cases, it will be necessary to follow up with genetic investigation to identify pathogenic mutations or expansions^3^. 

### Physical and neurological examination

A complete physical examination should be performed, including vital signs, cardiovascular, gastrointestinal, and genitourinary evaluation. 

The neurological examination should attempt to investigate abnormalities that are common in bvFTD and which are not characteristic of psychiatric disorders. In particular, motor changes related to bvFTD, such as parkinsonism, signs of motor neuron disease (muscle weakness, deep tendon hyperreflexia, amyotrophy, fasciculations, dysphagia or dysarthria), primitive reflexes (such as grasp reflex), and oculomotor disorders (smooth pursuit, especially vertical gaze, and saccadic eye movements) should be assessed.

### Psychiatric assessment

Due to the early and prominent behavioral changes in bvFTD, patients and their families are more likely to seek psychiatric help and are often initially diagnosed with a PPD, despite the fact that most of them do not meet formal criteria for PPD according to the Diagnostic and Statistical Manual of Mental Disorders (DSM-5)^10^ ^,^ ^12^. Therefore, there is a need for multidisciplinary team work, including a psychiatrist with expertise in bvFTD assessment.

**Figure 1.**
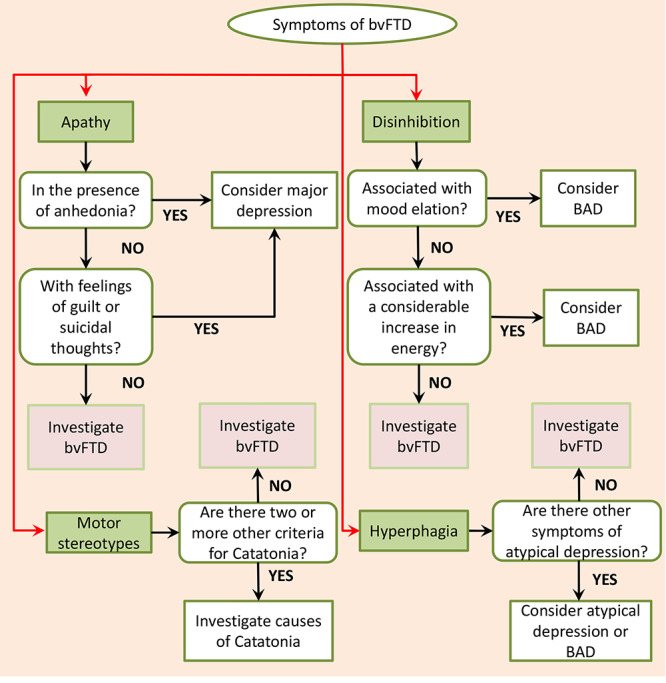
Suggested clinical approach to symptoms common to bvFTD and mood disorders/catatonia. Adapted from Ducharme et al. (2015)^10^.

The most common initial manifestations of bvFTD are apathy, loss of interest, lack of initiative and inactivity, with a clinical picture often misdiagnosed as major depression^13^. However, a sustained lowering of mood, a classic feature of depression, is uncommon in bvFTD^14^ (Figure 1). 

The loss of interest and/or ability to feel pleasure, recognized as anhedonia in major depressive disorders, may also mimic apathy in bvFTD. Nevertheless, apathy is independent of underlying motivational aspects and is characterized by impaired initiative or responsiveness in goal-directed behaviors^15^. It is crucial to recognize that apathy is not experienced as distressing or accompanied by dysphoria (Figure 1).

On the other hand, disinhibition, hypersexuality, irritability, hypoprosexia (i.e., difficulty in fixing attention on a stimulus), over-involvement in pleasurable activities, risk-taking behavior, inappropriate social conduct, compulsive behaviors, and reduced need for sleep can be confused with mania/hypomania of bipolar affective disorder (BAD)^16^. As with bvFTD patients, bipolar patients in mania can be jocular and inappropriate at times, but a permanently exalted mood, with obvious grandiosity and euphoria, present in BAD, are not common features of bvFTD^3^. Also, an obvious increase in energy, common in mania, does not usually accompany patients with bvFTD^17^ (Figure 1). Finally, BAD is classically characterized by its episodic manifestation, with cognitive and functional recovery after crises. Even though severe cases of BAD can progress with cognitive decline and chronic states, the cognitive impairment is not as profound or notoriously progressive as in bvFTD^18^. Nonetheless, it is a complex differential diagnosis and BAD was the second most frequently given diagnosis in a series of bvFTD patients, of whom almost 50% were diagnosed with a PPD before bvFTD was finally recognized^3^. Also, in a study conducted by the research group in which history of major depression was nor investigated, BAD was the most common diagnosis among the 36.9% of patients with a psychiatric diagnosis prior to bvFTD^19^.

Psychotic symptoms are not uncommon manifestations of bvFTD, appearing in about 10-20% of patients^20^, despite this not being considered a core symptom in the diagnostic criteria. Therefore, those symptoms can be easily confused with psychotic symptoms of schizophrenia or other primary psychotic disorders, particularly in those with *C9orf72* gene expansions^21^. The emotional blunting displayed by bvFTD patients is akin to the one classically described by Bleuler in the “Group of Schizophrenias”^22^, a cluster of symptoms later categorized in 1974 as part of the negative presentation of the disease^23^. A relevant feature for the differential diagnosis in this context is the age of onset of symptoms, since schizophrenic disorders mostly appear in early adulthood. In addition, schizophrenia does not have a progressive worsening pattern^24^.

Patients with bvFTD may exhibit motor stereotypies, mannerisms, negativism, or mutism. Such manifestations may refer to a picture of catatonia associated with another mental disorder, including BAD and depression. However, if more than two diagnostic criteria for catatonia are present, causes of catatonia should be investigated before assuming a compatibility of symptoms with dementia^25^ (Figure 1). 

Finally, although compulsive behaviors and collectivism are frequent presentations, confusion with the diagnosis of obsessive-compulsive disorder is not common^3^.

## COGNITIVE EVALUATION

### Brief cognitive tests

Cognitive screening tests are a major part of a complete clinical evaluation, and several instruments are routinely used. 

The Mini-Mental Status Examination (MMSE) proved to be a poor test to differentiate bvFTD from PPD, since patients with suspected bvFTD often perform normally, at least in the initial stages. The Montreal Cognitive Assessment (MoCA) has proven to be a better instrument than the MMSE for screening bvFTD patients, but its value for differentiating bvFTD from PPDs is still unknown^26^.

Executive function screening tests can depict executive impairment in different types of dementia and psychiatric disorders. However, the Frontal Assessment Battery (FAB) has already failed in differentiating bvFTD from PPD^27^. On the other hand, INECO Frontal Screening (IFS) has already been shown to be useful in distinguishing bvFTD and major depression or BAD and may be an important tool^28^. In a recent Brazilian study, the Addenbrooke Cognitive Examination-Revised (ACE-R) displayed good accuracy for the differential diagnosis between bvFTD and mild dementia due to AD^29^.

### Neuropsychological and social cognition evaluation

A neuropsychological examination is important especially in cases where cognitive decline is subtle or questionable, or in patients with premorbid high cognitive performance. Nonetheless, even in neuropsychological evaluation, performance on executive tasks can be within normal range. In such cases, it is important to register qualitative findings, such as the strategies used, patterns and repetitions, inflexibility, and impulsivity.

Social cognition tests display the best accuracy in diagnosing bvFTD. These tasks generally assess the comprehension of social rules, behavioral appropriateness to context, and emotional processing, particularly emotion recognition^30^.

Mini-SEA (Mini-Social Cognition & Emotional Assessment) is a neuropsychological battery aiming to evaluate the impairment of social and emotional cognition. Mini-SEA is composed of two subtests: the Faux-Pas test (“gaffes” test), which evaluates Theory of Mind, comprehension of rules, empathy, among others - and the Facial Emotion Recognition Test (FERT)^31^. Mini-SEA presents a good diagnostic accuracy to distinguish bvFTD from AD^32^, and the FERT has proven useful in differentiating bvFTD and late-life depression^33^ ^,^ ^34^. 

There are other tools available to assess social cognition, including the Reading the Mind in the Eyes Test (RMET), besides the Theory of Mind (ToM) task with 15 stories (TOM-15) and The Strange Stories Test. When compared with PPD, patients with bvFTD score worse on a ToM task (reading the mind in the eyes, RMET) than patients with bipolar disorder^35^.

## STRUCTURAL AND FUNCTIONAL NEUROIMAGING

Neuroimaging examination is a mandatory part of any investigation of suspected bvFTD in patients with psychiatric manifestations in adulthood. Despite often being an insufficient test in distinguishing bvFTD and PPD, structural neuroimaging (magnetic resonance imaging-MRI or computed tomography-CT) should always be requested and should include other functional imaging tests, such as positron emission tomography (PET) and single-photon emission tomography (SPECT)^36^ ^,^ ^37^.

The presence of anterior cortical atrophy on MRI or CT is critical for the diagnosis of probable bvFTD. The frontal lobe regions that are commonly affected include the orbitofrontal cortex, medial and lateral prefrontal cortices, and anterior cingulum, along with atrophy of the adjacent insular cortex. Imaging studies have shown that atrophy of the frontal and anterior temporal regions is related to the behavioral symptoms seen in bvFTD, including apathy, disinhibition, loss of empathy, and aggression^38^.

The results of MRI and FDG-PET have been shown to be useful in differentiating healthy individuals from individuals with bvFTD, as well as from patients with other neurodegenerative diseases^39^. However, the presence of hypometabolism appears to be of limited practical application when used in cohorts of adult-onset behavioral changes. Nevertheless, in some dubious cases, FDG-PET may be helpful in the exclusion of neurodegenerative etiologies or in showing metabolic changes in bvFTD subjects who do not present frontal and anterior temporal lobe atrophy on MRI or CT^40^.

## CEREBROSPINAL FLUID (CSF) BIOMARKERS

The use of CSF biomarkers to differentiate bvFTD from PPD is not yet available in clinical practice and its application has been limited to research. However, initial results using the neurofilament light chain as a degeneration marker to differentiate bvFTD from PPD are promising^41^.

For now, the CSF biomarkers used in practice are those used to rule out AD. Isolated increase in CSF total tau with no reduction in CSF amyloid-β42 is supportive of a diagnosis of bvFTD, since CSF amyloid-β42 in definite bvFTD was found to be normal, while CSF total tau levels are commonly increased, as a marker of neurodegeneration^41^ ^,^ ^42^.

## GENETICS

Around 30-50% of patients with bvFTD have a positive family history, affecting several people in the same family in consecutive generations. An autosomal dominant pattern of inheritance is found in 10-27% of all FTD cases, named genetic FTD^43^. 

Genetic FTD arises from a single genetic alteration or variant, known as a pathogenic gene mutation or expansion. About 80% of genetic FTDs are caused by one of three abnormalities: *MAPT* (microtubule associated protein tau), *C9orf72* (chromosome 9 open reading frame 72) and *GRN* (granulin)^44^. Cases are rarely caused by other pathogenic mutations, such as *TARDBP*, *VCP*, *CHMP2B*, *SQSTM1*, *UBQLN1* and others^45^.

Visual hallucinations and delusions occur in up to 25% of patients with *GRN* mutation during the course of the disease and can also be the first symptom. GRN mutations have also been found in patients with bipolar disorder that evolved into bvFTD^46^ ^,^ ^47^.

In patients carrying *C9orf72* expansion the differential diagnosis between bvFTD and PPD becomes even more challenging. In this group, psychotic symptoms are present in up to 56% of cases, and reports indicate that diagnoses of schizophrenia, BAD, or obsessive-compulsive disorder may arise years before the onset of dementia^21^ ^,^ ^48^. 

Distinguishing bvFTD from PPD remains a great challenge in clinical practice. There is a dramatic overlap of psychiatric symptoms in the absence of reliable biomarkers.

**Figure 2.**
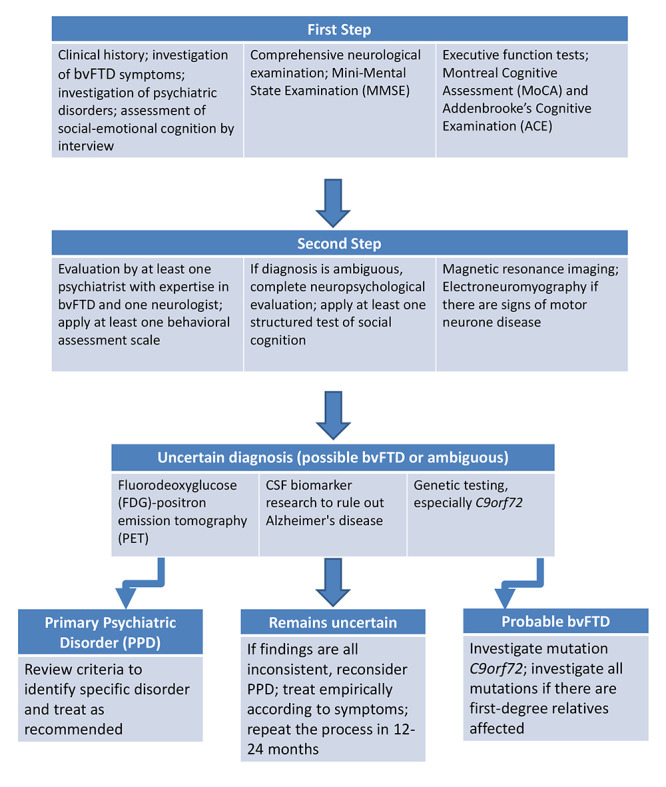
Algorithm to approach the differential diagnosis of bvFTD and PPD. Based on Ducharme et al (2020)^49^.

To summarize, careful history-taking is recommended, paying special attention to psychiatric symptoms, specifying the date of onset and the clinical course. The neurological evaluation should check signs of parkinsonism, oculomotor changes, and motor neuron disease. Evaluation of the patient by a multidisciplinary team is required, with the presence of a knowledgeable neurologist and a psychiatrist with expertise in bvFTD. The patient should be interviewed using the DSM-5, since most patients with bvFTD do not meet the criteria for PPD according to the manual. Cognitive screening tests should be used, especially the MoCA and the ACE-R, since the MMSE scores are often normal in the early stages of bvFTD. Executive function assessment tools may be useful in some cases. Structural neuroimaging tests are not sufficient to differentiate bvFTD from PPD, although they are necessary in any suspected patient. Functional examinations, especially FDG-PET, can help to suggest or to rule out neurodegenerative etiologies in more ambiguous cases. CSF examination has not yet established its usefulness in clinical practice, except to rule out AD, but neurofilament light chain is a promising biomarker. In the genetic assessment, particular attention is given to *C9Orf72* expansion carriers, since their phenotypes are the ones that are most likely to be confounded with PPD (Figure 2).
